# Characterization of Properties and Kinetic Analysis of Unsaturated Polyester Resin Synthesized from PET Alcoholysis Waste

**DOI:** 10.3390/polym17060820

**Published:** 2025-03-20

**Authors:** Ruixiang Wang, Hongliang Zhang, Jingshuang Liu, Tongjun Wei

**Affiliations:** 1 Key Laboratory for Special Area Highway Engineering of Ministry of Education, Chang’an University, Xi’an 710064, China; lx1297chw@163.com; 2 Guangxi Pingna Expressway Co., Ltd., Nanning 530022, China; ljs123fuj@sina.com; 3 Shandong Jiaofa Engineering Design Consulting Co., Ltd., Dongying 257100, China; 17865924232@139.com

**Keywords:** polyethylene terephthalate, alcoholysis, unsaturated polyester resins, properties

## Abstract

Recycling and reutilization of waste PET through alcoholysis has been a prominent focus of current research. However, the alcoholysis process is reversible, leading to the generation of oligomeric waste byproducts. To further utilize these wastes, this paper processed oligomeric waste derived from various alcoholysis systems to synthesize unsaturated polyester resins (UPRs). The fundamental characteristics, mechanical properties, and curing processes of synthesized UPRs were analyzed based on GPC, FTIR, TG, tensile testing, DMA, and DSC tests. The results indicate that wastes were successfully synthesized to UPRs. The UPRs synthesized from ethylene glycol (EG) and diethylene glycol (DEG) possess more complex compositions; among these, the UPR synthesized from EG exhibited higher thermal stability, whereas the UPR synthesized from DEG showed a broader molecular weight distribution and a lower glass transition temperature (*T_g_*). In addition, the UPR synthesized from DEG exhibited a remarkably high elongation at break (>180%), potentially attributed to its long molecular chains. Regarding curing characteristics, UPRs obtained from DEG and propylene glycol (PG) exhibited slower curing rates and demanded higher activation energies. Moreover, the curing processes of UPRs could be well described by the Sesták–Berggren autocatalytic model.

## 1. Introduction

Polyethylene terephthalate (PET) possesses excellent mechanical properties, chemical resistance, and stability [1,2], and is the most widely used polyester thermoplastic polymer in the world, with an annual global consumption exceeding 24 million tons [3,4]. However, PET is difficult to degrade under natural conditions, leading to a large amount of white pollution and increasingly severe environmental problems [5]. Therefore, recycling and reusing PET has become a pressing issue to be addressed. Generally, the recycling methods of PET can be divided into four categories [6]: direct recycling back to the factory (re-extrusion), mechanical processing followed by further utilization (mechanical), depolymerization of raw materials to synthesize new substances (chemical), and obtaining heat through combustion (energy recovery). Although direct recycling and mechanical treatment methods are cost-effective, they suffer from issues such as poor quality and low added value [7]. Compared to the original PET products, products obtained after further processing post-recycling exhibit various defects, including poor durability and inferior mechanical properties [8]. Furthermore, the combustion of PET generates harmful substances such as carbon monoxide and acetaldehyde, which may cause secondary pollution. As for chemical recycling methods, they allow for the adjustment of depolymerizing agent types to obtain different products, enabling the conversion of recycled products into raw materials for production. This makes chemical recycling an effective and advanced method based on the concept of atomic economy [9].

Currently, chemical recycling methods for PET generally include hydrolysis, alcoholysis, and amineysis [10]. Among these, alcoholysis is one of the commonly used methods for PET chemical recycling due to its relatively mild reaction conditions, lower solvent volatility, and high-value-added products [11,12,13]. Depending on the type of alcoholysis agent used, different monomers can be obtained during the alcoholysis process, which are subsequently utilized in the synthesis of unsaturated polyester resins, polyurethanes, alkyd resins, and other products [7]. Unsaturated polyester resins (UPRs), typically synthesized through the esterification polycondensation of diols and dicarboxylic acids, are thermosetting resins with significant commercial value, exhibiting outstanding mechanical properties, chemical resistance, and low cost, and are widely applied in industries such as the automotive and construction industries. The products of PET alcoholysis are diols, such as bis(2-hydroxyethyl) terephthalate (BHET) and bis(2-hydroxyphenyl propionate) (BHPT). These products can acquire different characteristics by adjusting the type of alcoholysis agent, resulting in UPRs with superior performance compared to conventional UPRs. Zhang et al. [7] conducted alcoholysis of PET using a mixture of propylene glycol (PG) and diphenylsilanediol (DDS) and synthesized a UPR with excellent flame-retardant properties based on the alcoholysis products. Bórquez-Mendivil et al. [14] further introduced SiO_2_ into the synthesis process based on PG alcoholysis, thereby enhancing the hardness and Young’s modulus of the resulting resin. García et al. [15] performed alcoholysis of PET with ethylene glycol (EG), utilized the obtained BHET to prepare a UPR, and reinforced it with corn stalk fibers. The results indicated that while the mechanical strength of the resin decreased, its water absorption capacity increased (ranging from 0.6% to 2.56%). Ramírez-Palma [16] compared the performance of UPRs synthesized from two different diol alcoholysis products, BHET and BHPT. The experiments demonstrated that BHET could enhance the tensile strength of UPR, whereas UPRs based on BHPT exhibited better compatibility with styrene.

**Figure 1:**
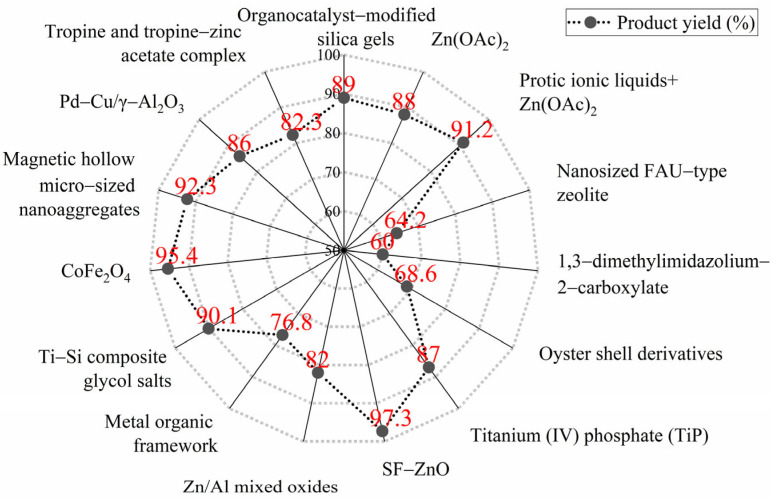
Monomer yields of different catalysts in the PET alcoholysis system using EG [18,19,20,21,22,23,24,25,26,27,28,29,30,31,32].

From the above discussion, it is evident that there is extensive research on synthesizing UPR from PET-based alcoholysis monomers. However, the alcoholysis reaction is reversible, preventing the complete conversion of PET into monomers and resulting in the presence of some dimers and oligomers within the system [17]. These oligomers typically possess higher molecular weights and longer molecular chains, making them unsuitable for use as monomers in subsequent processes. Many studies have explored methods to increase monomer yield, proposing approaches such as the use of more efficient catalysts, improvement of the alcoholysis process, and the addition of larger quantities of alcoholysis agents. These strategies aim to shift the reaction equilibrium toward the production of monomers. Although certain progress has been achieved, attaining a 100% monomer yield remains unfeasible. As illustrated in Figure 1 [18,19,20,21,22,23,24,25,26,27,28,29,30,31,32], the highest monomer yield in PET alcoholysis systems using ethylene glycol (EG) as the alcoholysis agent currently reaches 97.3%, which is a 21.6% improvement over the typical industrial monomer yield of 80%. However, this method is cost-intensive and thus not suitable for broader application. In addition to EG alcoholysis, other alcoholysis agents such as propylene glycol [33], diethylene glycol [34], and neopentyl glycol [17] also encounter the challenge of not achieving 100% monomer yield.

**Figure 2:**
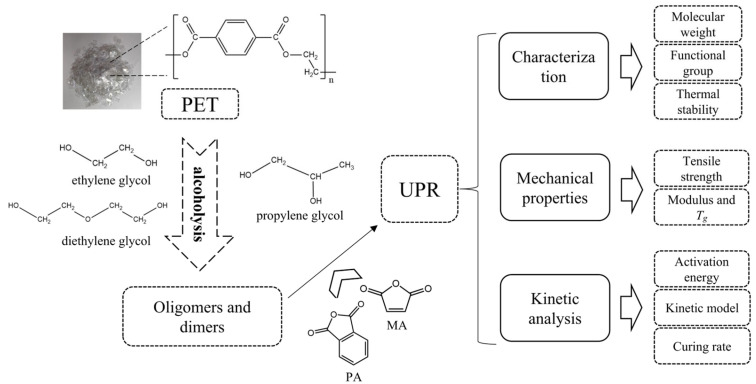
Diagram of research methodology.

These oligomers, resulting from depolymerization with diols, possess hydroxyl groups at both ends and can therefore be considered as a type of long-chain diol. In the synthesis of UPR, extending the length of the main chain can enhance the toughness. Although this may reduce its strength, the oligomers derived from PET alcoholysis contain numerous benzene rings, whose rigidity can partially offset the decrease in strength associated with longer chains. Within current alcoholysis systems, EG remains the predominant glycolytic agent, while alternative alcohols such as PG, DEG, and neopentyl glycol (NPG) have received comparatively limited research attention. This investigation systematically selected the three most extensively studied and commonly utilized glycolytic agents (EG, PG, and DEG) to synthesize UPRs from their respective oligomeric byproducts. The fundamental characteristics, mechanical properties, and curing properties of the synthesized UPRs were investigated with the aim of providing a feasible method for recycling these alcoholysis waste products. This strategy not only enhances the recycling efficiency of PET but also demonstrates potential for cost reduction in UPR production through waste valorization. The research methodology is illustrated in Figure 2.

## 2. Materials and Methods

### 2.1. Raw Materials

Waste PET was obtained from recycled plastic bottles, which were shredded into approximately 1 cm × 1 cm pieces. The shredded PET was washed with alcohol and water to remove surface contaminants and then dried in a drying oven at 60 °C for 6 h. EG, PG, DEG, ethyl acetate, ethanol, phthalic anhydride (PA), maleic anhydride (MA), hydroquinone, and benzoyl peroxide (BPO) were all purchased from Shanghai McLin Biochemical Technology Co., Ltd., Shanghai, China. All reagents were of analytical grade and used without further purification.

### 2.2. Preparation of Oligomers and UPRs

(1) Preparation of oligomers

**Figure 3:**
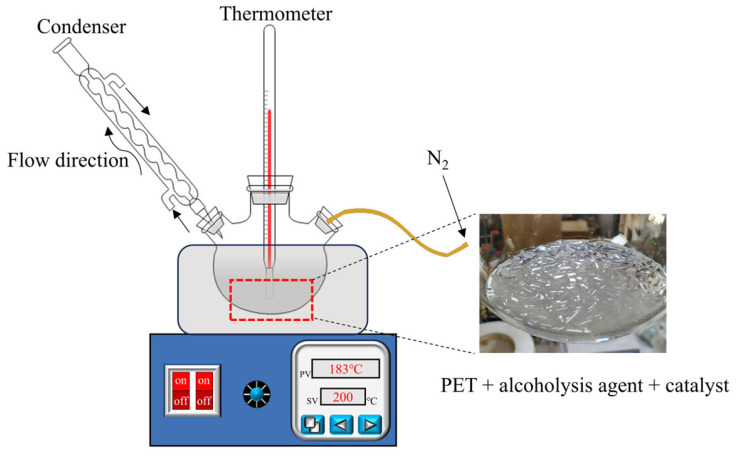
Diagram of the alcoholysis apparatus.

Alcoholysis agents were added in a molar ratio to PET of 1:5, and Zn(OAC)_2_ was used as a catalyst at 0.1% of the PET mass. The reaction was carried out in the apparatus illustrated in Figure 3 until the PET was completely dissolved. Due to the different alcoholysis products obtained from the three types of alcoholysis agents, the purification processes for the monomers also varied.

For the EG alcoholysis system, the reaction mixture was washed with excess boiling water and filtered. Once the filtrate cooled to approximately 60 °C, vacuum filtration was performed to obtain the residue, which was then dried in an oven for over 12 h to obtain the bis(2-hydroxyethyl) terephthalate (BHET) oligomer. In the PG alcoholysis system, after the reaction was completed, the mixture was distilled at 190 °C. Subsequently, water and ethyl acetate were added to the reaction mixture, causing the precipitation of bis(2-hydroxyphenyl propionate) (BHPT) and its oligomers. The difference in solubility between the monomers and oligomers in certain solvents was exploited by adding a small amount of ethanol to dissolve the BHPT monomer, leaving behind the BHPT oligomer. For the DEG alcoholysis system, the resulting reaction mixture was distilled at 250 °C to remove the DEG. The remaining liquid was then crystallized in a low-temperature environment and filtered, yielding the residual liquid phase as the oligomer.

(2) Synthesis of UPR

A two-step method was employed to synthesize UPR, which is obtained through the polycondensation reaction of diols and dicarboxylic acids. Initially, the oligomers produced from alcoholysis and a saturated diacid (PA) were added to a flask, and the mixture was maintained at 190 °C for approximately 0.5 h. Subsequently, the temperature was reduced to around 170 °C, and an unsaturated acid (MA) was introduced. During this stage, the water generated from the reaction was promptly removed, and the acid value of the system was continuously monitored. Once the acid value decreased to approximately 60 mg KOH/g, it indicated that the esterification reaction was nearly complete. The system was then heated to above 200 °C to complete the polycondensation reaction. Heating ceased when the acid value dropped to around 25 mg KOH/g. After cooling, a specific amount (about 20–30wt% of UPR) of styrene was added to dilute the mixture, ensuring that the final product possessed adequate flowability for casting and molding. Additionally, during the dilution process, 0.2 wt% of hydroquinone was incorporated to prevent the UPR from undergoing self-polymerization, which could adversely affect subsequent studies. The UPR samples obtained from the oligomeric products derived from EG, PG, and DEG alcoholysis were designated as UEG, UPG, and UDEG, respectively.

(3) Preparation of UPR casting body

Benzoyl peroxide (BPO) was selected as the initiator, with an addition amount of 2 wt% relative to the mass of the UPRs [35]. After uniformly mixing the initiator with the resin prepared in step (2), the mixture was poured into the corresponding molds and cured at 20–25 °C for over 24 h. Subsequently, the cured UPRs were demolded to proceed with further testing.

### 2.3. Test Methods

(1) Gel permeation chromatography (GPC)

The molecular weight and distribution of the synthesized UPR were determined using GPC. An Agilent 1260 (purchased from Shanghai Shanfu Electronic Technology Co., Ltd., Shanghai, China) chromatographic system equipped with three tandem columns was employed for the analysis. The measurements were conducted at a temperature of 35 °C with a flow rate of 1 mL/min. Approximately 5 mL of each synthesized resin was dissolved in tetrahydrofuran (THF) and subsequently injected into the chromatographic system. Calibration was performed using linear polystyrene standards.

(2) Fourier transform infrared spectrometry (FTIR)

The functional groups of UPRs were characterized using an IRTracer 100 infrared spectrometer (purchased from Guangdong Shengze Technology Co., Ltd., Luoding, Guangdong, China). Measurements were performed in attenuated total reflectance (ATR) mode by placing a small amount of resin sample onto the contact surface of the ATR crystal to ensure uniform coverage of the contact area. The spectral resolution was set to 4 cm^−1^, and the scanning range extended from 4000 to 400 cm^−1^.

(3) Thermogravimetric analysis (TG)

Thermogravimetric analysis (TG) was conducted using a TA-STD650 (purchased from Shenzhen Taili Instrument Co., Ltd., Shenzhen, China) thermal analyzer. Approximately 10–15 mg of the sample was put into an alumina crucible and heated from room temperature to 800 °C at a heating rate of 10 °C/min under a nitrogen atmosphere. The temperature reproducibility of the instrument was ±1 °C.

(4) Tensile test

The samples were molded in accordance with GB/T 2567-2021 [36] and conditioned at room temperature with a relative humidity below 75% for 48 h. Tensile testing was performed at a temperature of 25 °C with a strain rate of 10 mm/min. Three specimens were tested in each group, and the final results were averaged, with the error controlled within 15%.

(5) Dynamic mechanical analysis (DMA)

DMA was conducted using a TA-QMA Q800 (purchased Shenzhen Xinyichuang Technology Co., Ltd., Shenzhen, China) instrument from TA Instruments, equipped with a double cantilever fixture. The sample dimensions were specified as 50 mm × 10 mm × 4 mm. The temperature was increased from –30 °C to 150 °C at a heating rate of 3 °C/min. The testing frequency was set to 1 Hz, and the amplitude was maintained at 0.1% of the strain. The glass transition temperature was determined in accordance with ASTM D7028-07 [37].

(6) Differential scanning calorimetry (DSC)

Following the operational procedures outlined in GB/T 19466.1-2004 [38], a 1.5 wt% initiator was added to the synthesized UPRs and rapidly stirred to ensure uniform mixing. Subsequently, 5 mg to 10 mg of the sample, accurate to 0.1 mg, was placed into an aluminum crucible. Under a nitrogen atmosphere, thermal analysis was performed at four heating rates, 5 °C/min, 10 °C/min, 15 °C/min, and 20 °C/min, with a gas flow rate of 50 mL/min.

## 3. Results and Discussion

### 3.1. Characterization of UPRs

#### 3.1.1. Distribution of Molecular Weight

**Figure 4:**
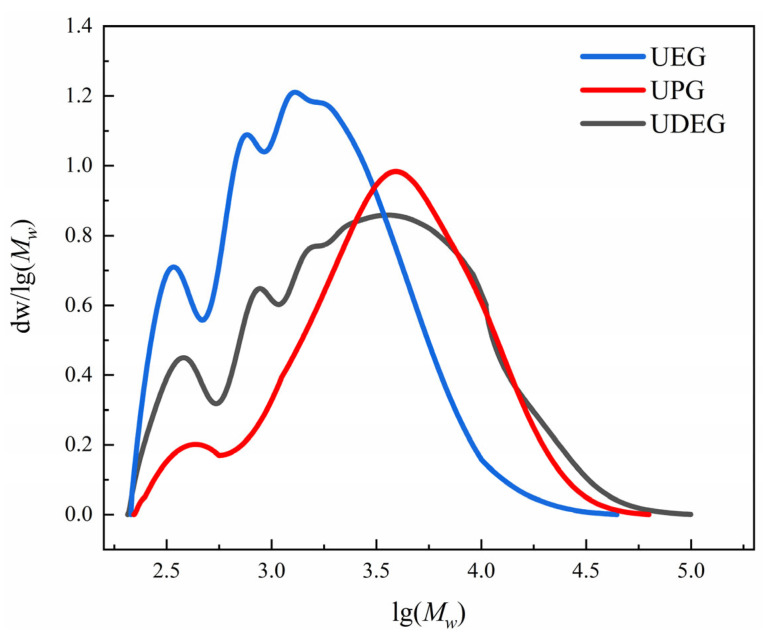
Distribution of molecular weights of UPRs.

The GPC test results for each UPR are presented in Figure 4. It can be observed that all three resins exhibit peaks at lower molecular weights, which may indicate the presence of some residual depolymerized monomers during the preparation of the oligomers. These residual monomers likely react with anhydrides in subsequent synthesis steps to form polymers. The curves for UEG and UDEG show noticeably more peaks compared to UPG, and the peak positions for UEG and UDEG correspond to approximately the same molecular weights, although the peak intensities differ. This phenomenon may be attributed to the mutual interconversion of EG and DEG under high-temperature conditions. Specifically, in an acidic environment above 150 °C, EG molecules undergo dehydration, condensation, and etherification to form DEG. Conversely, DEG can react with water molecules under the same conditions, leading to the cleavage of ether bonds and the regeneration of EG. Therefore, the alcoholysis products obtained from these two systems may contain identical substances. For UPG, the molecular weight distribution curve exhibits fewer fluctuations, which may be due to the more complex structure of PG compared to EG. Etherification reactions involving PG require higher temperatures and longer reaction times under high-temperature conditions. Additionally, the alcoholysis time for PG is much shorter than that for EG, resulting in a more uniform alcoholysis product in UPG.

To further analyze the molecular weights of the three UPRs, the number-average molecular weight (*M_n_*), weight-average molecular weight (*M_w_*), z-average molecular weight (*M_z_*), and polydispersity index (PDI) were calculated. The results are presented in Table 1. Generally, the low molecular weight fraction contributes more significantly to *M_n_*, indicating that *M_n_* is more reflective of the number of molecular chains in the polymer. In contrast, *M_w_* emphasizes the weight of individual molecular masses, placing greater emphasis on the high-molecular-weight components. According to the results in the table, the *M_w_* of UEG, UPG, and UDEG progressively increases, indicating that the molecular weight of the alcoholysis agent directly affects the molecular weight of the synthesized UPR. However, the *M_n_* of resin UPG is higher than that of UDEG, suggesting that UDEG contains a greater proportion of high-molecular-weight molecules. Additionally, the calculation of *M_z_* further emphasizes the weight of individual molecular masses based on *M_w_*, making it more susceptible to the influence of large molecules. It can be observed that the *M_z_* values of all resins are significantly greater than their *M_w_*, indicating that all UPRs contain a portion of ultra-high-molecular-weight molecules, which is beneficial for enhancing the mechanical properties. The polydispersity index, obtained by dividing *M_w_* by *M_n_*, reflects the degree of molecular weight distribution of the polymer. The PDI of UDEG is significantly higher than those of UEG and UPG, which is related to the aforementioned decomposition of DEG into EG under high-temperature acidic conditions. Although EG can also polymerize to form DEG, both the decomposition and polymerization reactions produce fewer products. An increase in *M_w_* enhances the weighting of molecular mass, resulting in a greater number of macromolecules in UDEG exhibiting higher *M_w_*. Consequently, this intensifies the degree of molecular weight distribution.

#### 3.1.2. Analysis of Functional Groups

**Figure 5:**
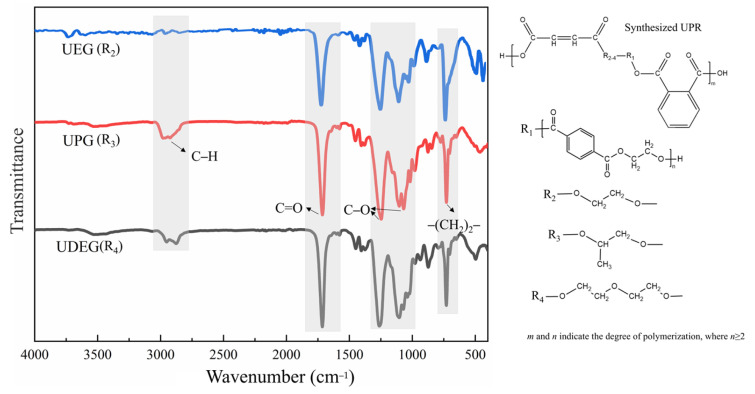
Infrared spectra of UPRs.

The FTIR spectra of the three UPRs are presented in Figure 5, where R_1_, R_2_, R_3_, and R_4_ represent the structures of PET, EG, PG, and DEG, respectively. It is evident that all resins exhibit similar absorption peaks. Absorption peaks at 2979 cm^−1^ and 2876 cm^−1^ are attributed to the symmetric and asymmetric stretching vibrations of C–H bonds, respectively. A strong absorption peak near 1710 cm^−1^ is observed, which is caused by the C=O stretching vibrations in fatty acid esters. In the case of unsaturated polyesters, conjugation between the unsaturated double bonds and carbonyl groups may occur, resulting in a redshift of the C=O absorption peak. Additionally, strong absorption peaks are present around 1250 cm^−1^, 1100 cm^−1^, 1060 cm^−1^, and 720 cm^−1^ in UPRs, corresponding to the symmetric and asymmetric stretching vibrations of C–O bonds and the in-plane rocking vibrations of –(CH_2_)_2_– groups, respectively. Besides these prominent absorption peaks, weak absorption peaks are also observed at 1583 cm^−1^, 1452 cm^−1^, 870 cm^−1^, and 779 cm^−1^, indicating the presence of benzene rings. These weak peaks correspond to the stretching vibrations of conjugated C=C bonds in the benzene rings and the out-of-plane stretching vibrations of C–H bonds, respectively. Furthermore, no characteristic absorption peaks of maleic anhydride are detected at 1780 cm^−1^ and 1870 cm^−1^, indicating that maleic anhydride has been fully incorporated into the resin matrix. The weak absorption peak observed at 1640 cm^−1^ (corresponding to C=C) also confirms the introduction of unsaturated bonds.

#### 3.1.3. Thermal Stability

**Figure 6:**
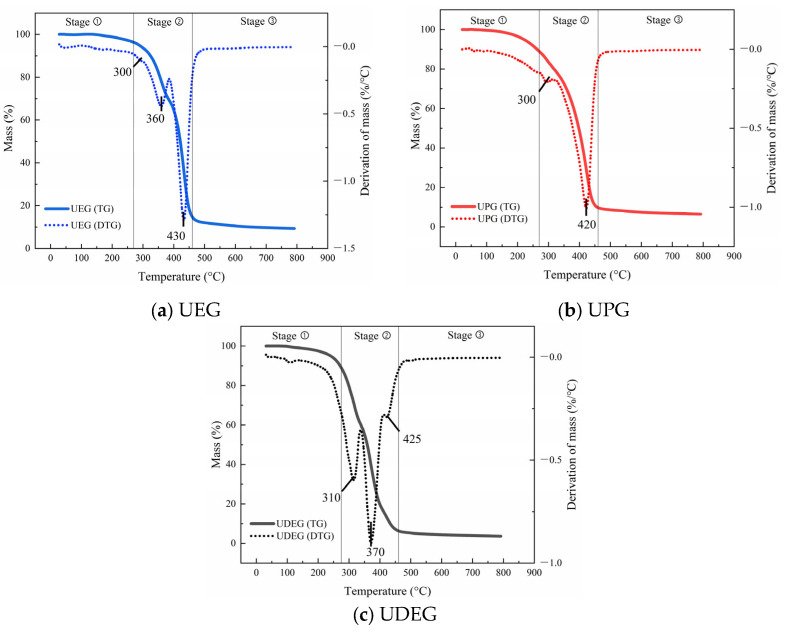
TG test results of UPRs.

The TGA results for the synthesized UPRs are presented in Figure 6. The thermal degradation of UPR under high-temperature conditions can generally be divided into three stages: dehydration loss (120–275 °C), statistical rupture of polyester and polystyrene chains (275–460 °C), and carbonization (460–600 °C) [39]. As shown in the figure, during the first stage, the mass loss of the UPRs is relatively small. When the temperature reaches 275 °C, the mass losses of the three UPRs are 4.013%, 10.997%, and 11.815%, respectively. Compared to UEG, UPG and UDEG exhibit greater dehydration mass loss, which may be attributed to the higher viscosity of these two resins. During the pre-esterification process, water molecules generated from carboxyl and hydroxyl groups are more difficult to eliminate from the system and further penetrate into the synthesized resin, resulting in a higher content of water molecules in the resin. In the second stage, UEG, UPG, and UDEG lose 81.185%, 82.674%, and 78.420% of their mass, respectively. In this stage, UDEG exhibits the least mass loss, which is due to the introduction of DEG into UPR, facilitating carbon formation. Guo et al. have demonstrated [40] that DEG can to some extent enhance the flame retardancy of UPR. Among the three synthesized UPRs, UEG may experience slightly less mass loss than UPG in this stage due to the presence of a small amount of DEG in the system. Generally, there is only one peak in the DTG curve of traditional UPRs, while the synthesized UPRs have multiple peaks, which could be related to the difference in the degree of polymerization of the raw materials used [7]. From the DTG curves, it can be observed that UPRs experience the fastest mass losses at 430 °C, 420 °C, and 370 °C, respectively. This phenomenon is related to the varying crosslink densities caused by different chain lengths. Resins with higher crosslink densities can form more compact and stable three-dimensional network structures, effectively enhancing the strength of molecular chains and thereby improving the thermal stability of the resin. Additionally, aside from the main peaks in the DTG curves, several minor peaks appear at other temperatures, indicating changes in the mass loss rates of UPRs near these temperatures. This could be attributed to the uneven chain length distribution in the synthesized UPRs. In the case of UPG, the chain length distribution is relatively uniform, resulting in minimal variation in the corresponding mass loss rates.

### 3.2. Mechanical Properties of UPR

#### 3.2.1. Results of Tensile Test

**Figure 7:**
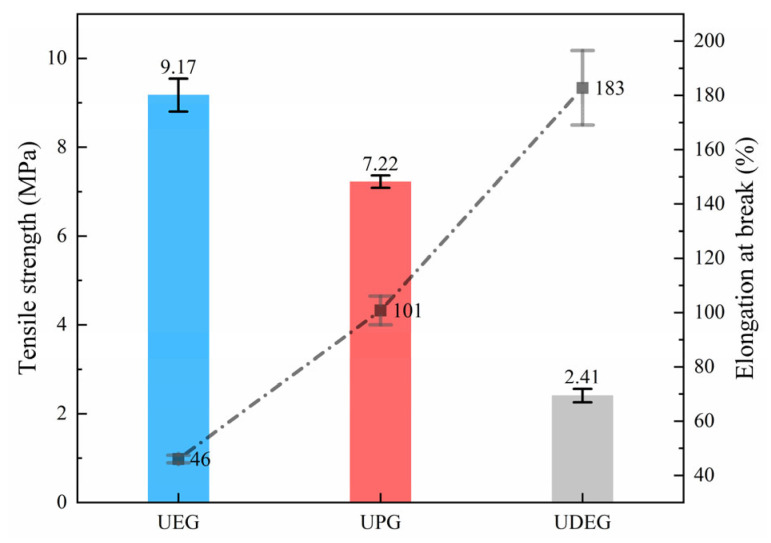
Tensile test results of UPRs.

The tensile results are shown in Figure 7. The data indicate that the tensile strength and elongation at break of the synthesized UPRs decrease and increase, respectively, with the increasing chain length of the alcoholysis oligomer. This suggests that chain length directly affects the mechanical properties of UPR. Longer molecular chains reduce the crosslinking density between molecules, granting the resin greater segmental flexibility and facilitating intermolecular movement. Consequently, the molecules have more space to move and rearrange under stress, resulting in higher extensibility. Conversely, the decreased crosslinking density leads to reduced tensile strength. Compared to neat UPRs (tensile strengths ≥ 40 MPa) [41], the synthesized UPRs exhibit significantly lower tensile strengths, with UDEG only reaching 2.41 MPa. This substantial reduction severely limits their practical application environments. The considerable decrease in tensile strength is attributed not only to the reduced crosslinking density but also to the uneven molecular weight distribution. In resin structures dominated by long chains, some low-molecular-weight segments may become weak points, thereby diminishing the overall strength of the resin. Although the reduction in tensile strength is substantial, all three synthetic resins exhibit high elongation at break, with the lowest value reaching 46%, more than four times that of traditional UPR (which has an elongation at break below 10%) [42]. This indicates that the synthesized UPR is more suitable for applications that require lower strength and greater deformation. Additionally, the incorporation of long-chain molecules may lead to a decrease in the chemical corrosion resistance of UPR, which should also be taken into consideration in its applications.

#### 3.2.2. Results of DMA

**Figure 8:**
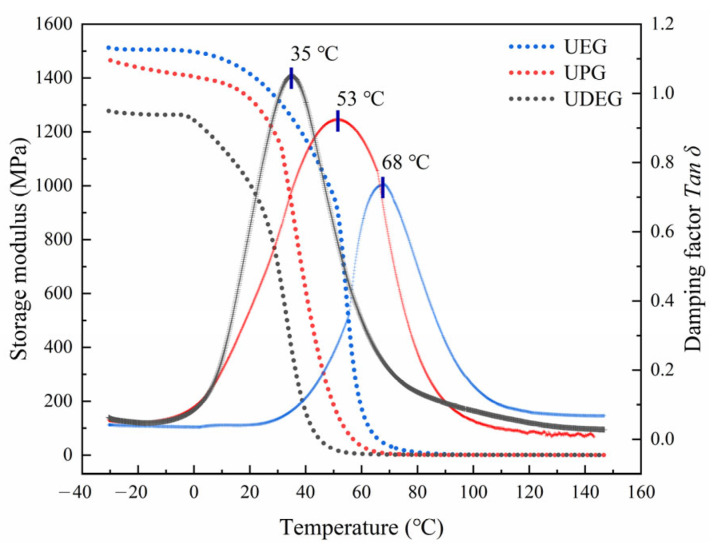
Storage modulus and damping factors of UPRs.

Dynamic mechanical analysis (DMA) can describe the structural effects in different polymer systems by observing changes in the viscoelastic properties of material, as shown in Figure 8. The storage modulus refers to the amount of energy stored elastically when the material undergoes deformation, reflecting the elastic characteristics of material. From the results depicted in the figure, it can be observed that the storage modulus of the UPRs decreases with increasing temperature. This decline is attributed to the enhanced thermal motion of the molecular chains at high temperatures, which reduces the mutual restrictions between molecular chains, making slip and rotation more facile. Additionally, the rise in temperature leads to an increase in the free volume of the resin, further enhancing the mobility of the molecular chains and thereby weakening the elastic properties. Furthermore, it is evident that UEG exhibits the highest storage modulus, while UDEG has the lowest. This difference may be due to the longer molecular chains of the oligomers obtained from the alcoholysis of DEG, as the presence of long chains facilitates the mutual movement of polymer chains. Moreover, the existence of long-chain molecules increases the distance between unsaturated bonds, resulting in a decreased crosslink density during the curing process, which further reduces the storage modulus. The damping factor (*Tanδ*) serves as an indicator for characterizing the viscoelasticity of materials, with its magnitude being directly proportional to the viscous behavior of material. The glass transition temperature (*T_g_*) of a material corresponds to the peak position of the damping factor curve. According to the results presented in the figure, UDEG exhibits the lowest *T_g_*, at only 35 °C, which implies that UDEG may be unsuitable for applications in high-temperature regions. The *T_g_* of traditional UPR is generally above 80 °C [43], and the UPRs synthesized from alcoholysis waste are quite different from them. This phenomenon could be attributed to the introduction of long-chain oligomers, which reduces the crosslink density on the backbone, weakens the polar interactions and hydrogen bonding between molecular segments, decreases the resistance to molecular motion of UPR, and enhances its ability to dissipate external energy. Consequently, this is manifested by an increased peak value of the damping factor and a shift toward lower temperatures. Additionally, the damping factor curve corresponding to UDEG is relatively broader, due to the presence of long-chain segments that allow for more molecular motion modes, indicating that the material possesses more relaxation modes. This is somewhat related to the broader molecular weight distribution of UDEG mentioned in Section 3.1.1. It is noteworthy that all UPRs exhibit only a single peak in *T_g_* curves, indicating the absence of other phases in three UPRs and that the cured products are homogeneous copolymers.

Furthermore, according to rubber elasticity theory, the rubber modulus is directly proportional to the crosslink density (*v_e_*) [44,45], which can be used to quantitatively estimate the changes in crosslink density [43]. Based on the DMA results, it can be assumed that all UPRs are in the rubbery state at 90 °C. Consequently, the *v_e_* for each UPR can be calculated using the formula presented in Equation (1), and the results are shown in Table 2.

(1) $$v_{e}=\frac{E_{r}}{3\phi RT}$$

where *E_r_* is the storage modulus in the rubbery region, *ϕ* is the front factor, obtained from the behavior of the model networks, and is set to 1 during calculations [46], *R* is the gas constant, *R* = 8.3145 J/(mol·K), and *T* is the absolute temperature.

According to the results presented in Table 2, UEG exhibits the highest crosslink density, which is consistent with its highest *T_g_*. However, although the *T_g_* of UPG is higher than that of UDEG, its crosslink density is slightly lower than that of UDEG. This could be attributed to the partial decomposition of DEG into EG during the alcoholysis of PET, resulting in the presence of oligomers with relatively shorter chain lengths in the system. These shorter-chain oligomers partially enhance the crosslink density of resin during the curing process. Nonetheless, the amount of EG produced in this system is relatively small [47], making it difficult to significantly influence the thermodynamic properties on a macroscopic scale. Consequently, the *T_g_* of the UPRs remains predominantly affected by the properties of the main alcoholysis products.

### 3.3. Kinetic Analysis of UPRs

The curing of UPR is a complex exothermic reaction in which monomers within the resin crosslink to form a network structure, which primarily determines its properties. Therefore, analyzing this process can provide valuable insights into the kinetics of UPR. This section analyzes the exothermic behavior of three synthesized UPRs during curing, based on DSC experimental results.

#### 3.3.1. Analysis of Curing Process

**Figure 9:**
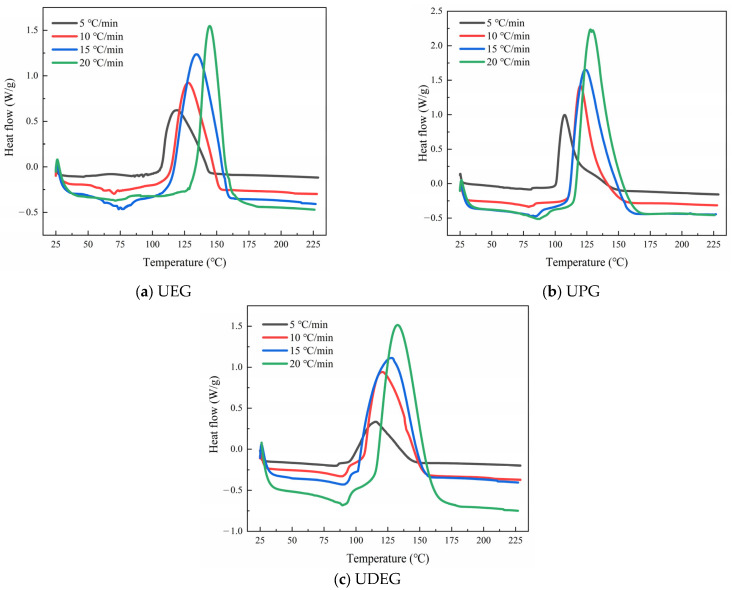
Exothermic curves of synthesized UPRs.

In this paper, a non-isothermal differential scanning calorimetry method was employed. The exothermic curves of UPRs are shown in Figure 9.

From Figure 9, it can be observed that all the exothermic peaks of UPRs shift toward higher temperatures as the heating rate increases. It is noteworthy that the exothermic curves of synthesized UPRs exhibit only a single exothermic peak, whereas the exothermic curves of traditional UPR display two distinct peaks due to the cross-polymerization of unsaturated polyester with styrene and the homopolymerization of unsaturated polyester. This indicates that the introduction of long-chain molecules has eliminated the homopolymerization process of unsaturated polyester [48]. Furthermore, the starting temperatures of the exothermic peaks for UPRs are 117.1 °C, 102.5 °C, and 99.6 °C, respectively. This is attributed to the increase in oligomer chain length, which leads to a reduction in styrene molecules as the viscosity of UPRs increases. Consequently, styrene radicals more readily polymerize with other styrene molecules and begin to release reaction heat at lower temperatures [48]. This similarly affects the peak temperatures of the exothermic peaks, as shown in Table 3. Table 3 presents the temperatures at peaks (*T_p_*) and the widths of peaks (*P_w_*) corresponding to the exothermic peaks of each UPR at different heating rates. It is evident that UDEG has the largest peak width, indicating the slowest curing rate. This is due to the generation of longer-chain saturated polyester through pre-esterification reactions during the preparation process. The longer the chain length of the saturated polyester, the stronger its spatial effect on the cross-polymerization of micro-gel particles, thereby hindering the polymerization rate of the UPR.

To further analyze the energy changes during the curing process of UPRs, the activation energy at different conversion rates can be calculated using the Starink method [49], as shown in Equation (2). In the curing kinetics of resin, it is assumed that the total area of the DSC exothermic curve is proportional to the total exothermic heat of the curing reaction. Based on this, the conversion rate α of resin, which represents the proportion of the resin that has reacted during the curing process relative to the total reactive portion, can be determined using Equation (3).

(2) $$\ln\left(\frac{\beta }{T^{1.92}}\right)=Const-1.0008\frac{E_{a}}{RT}$$

where *β* is the heating rate (°C/min), *T* is the absolute temperature (*K*), *Const* is a constant, *E_a_* is the activation energy (J/mol), and *R* is the gas constant, equal to 8.3145 J/(mol·K).

(3) $$\alpha =\frac{H\left(t\right)}{H_{0}}$$

where *H*(*t*) represents the total heat released from the initial moment to time t during the reaction, and *H*_0_ denotes the total heat released throughout the entire reaction process.

**Figure 10:**
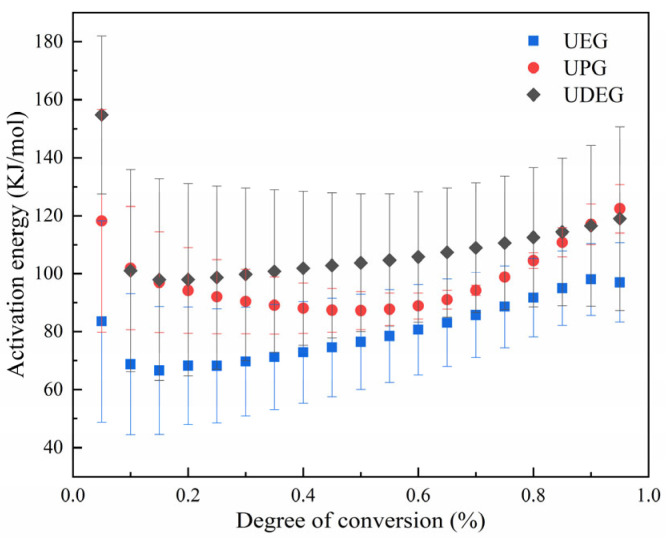
Activation energies of UPRs at various degrees of conversion.

Based on Equations (2) and (3), the activation energy of UPRs at different degrees of conversion was calculated, and is illustrated in Figure 10. It can be observed that the activation energy of UPRs undergoes a decrease from high to low during the initial stages of the reaction. This is because the polymerization reaction of UPR requires the initiator to decompose into free radicals, and the initiation process generally necessitates overcoming a high energy barrier, resulting in a higher activation energy to break the unsaturated double bonds and initiate the reaction. Once the reaction commences, the reaction system contains a high concentration of free radicals, making the reaction relatively easier and thereby reducing the activation energy. The data in the figure indicate that the activation energy increases as the reaction progresses further. This increase is attributed to the continuous polymerization of resin molecules and styrene, leading to an increase in molecular weight and viscosity, which restricts molecular movement. Additionally, the proliferation of crosslinking reactions requires more energy to overcome intermolecular resistance, resulting in an elevated activation energy. Furthermore, the activation energy of UDEG is consistently higher than those of UEG and UPG, indicating that the length of saturated chains in the UPR directly affects the curing reaction of the resin. Generally, longer chain lengths increase the viscosity of the resin, thereby limiting the movement of free radicals and other species within the resin system. Moreover, longer saturated molecular chains reduce the density of reactive sites in the system, decreasing the probability of effective collisions between reactants. These factors necessitate higher energy to overcome the molecular motion barriers during the reaction. Typically, when the activation energy varies with the degree of curing, it suggests a more complex reaction process, and the presence of competing reactions within the system can exacerbate deviations. During the entire curing phase, the variations in activation energy for UEG, UPG, and UDEG are 31.46 kJ/mol, 35.13 kJ/mol, and 56.80 kJ/mol, respectively. This indicates that the reaction within the UDEG resin system is relatively more complex, with more intense competition among reactants.

#### 3.3.2. Calculation of Kinetic Model

The curing process of UPR is a complex exothermic reaction, and the relationship between reaction rate and temperature can be described as follows:

(4) $$\frac{d\alpha }{dt}=K(T)f(\alpha )$$

where *f*(*α*) is the reaction model and *K*(*T*) is the rate constant.

(5) $$K(T)=A\exp(\frac{-E}{RT})$$

where *A* is the pre-exponential factor, while *E* stands for the apparent activation energy. *R* represents the gas constant, and *T* denotes the absolute temperature.

The function *f*(*α*) typically varies depending on the specific curing process involved. According to existing research, UPR exhibits a significant autocatalytic effect during the curing process [50,51]. Therefore, this section adopts the Sesták–Berggren (SB) autocatalytic model for calculations. The curing kinetic equation of the resin can be expressed as

(6) $$\frac{d\alpha }{dt}=\beta \frac{d\alpha }{dT}=\left[A\exp\left(-\frac{E}{RT}\right)\right]\alpha^{m}{\left(1-\alpha \right)}^{n}$$

where *β* denotes the heating rate, and *m* and *n* represent the reaction orders.

The experimental data were incorporated into the model using nonlinear multivariate regression. The model parameters were estimated by employing the Levenberg–Marquardt algorithm, and the results are presented in Table 4.

In the SB model, the pre-exponential factor *A* represents the collision frequency between reacting species in the system over time. According to the results presented in the table, UPG exhibits the lowest collision frequency. This might be related to the previously mentioned observation that during the alcoholysis process, a portion of the alcoholysis agents, such as EG and DEG, undergoes etherification or decomposition, thereby increasing the complexity of the alcoholysis products. Furthermore, the apparent activation energy *E* indicates the degree of difficulty in the curing reaction. UEG has the lowest apparent activation energy, which is close to that of UPG. This could be because in the reaction system of UPR, the apparent activation energy represents the combined effect of all possible steps during the curing process rather than the activation energy of a single step, unlike the activation energies at individual curing stages. According to the results shown in Figure 10, it is evident that in the curing degree range of 0.4–0.8, the activation energies of UEG and UPG are similar, thereby narrowing the overall activation energy levels of these two resins. The reaction order (*m* + *n*) reflects the curing rate of the resin system. Generally, a higher reaction order corresponds to a faster curing rate. Among the three UPRs, UEG exhibits the fastest curing rate, while UDEG is the slowest. This is consistent with the analysis results for their corresponding exothermic peak widths.

**Figure 11:**
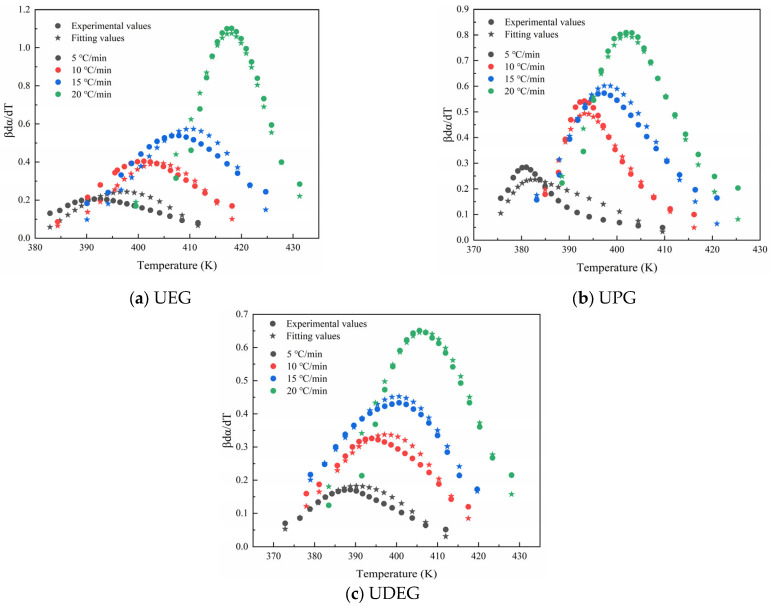
Comparison of SB model fitting values and experimental values.

To verify whether the fitted kinetic equations can accurately represent the actual curing process, the fitted results were compared with the experimentally obtained data, as shown in Figure 11. It can be observed that the fitted results closely match the experimental data, indicating that the SB autocatalytic model can effectively describe the curing process of synthesized UPRs.

## 4. Conclusions

This paper synthesized high-toughness UPRs utilizing incompletely alcoholysed PET waste from various alcoholysis systems. The fundamental properties of the synthesized UPRs were characterized through GPC, FTIR, and TG. Subsequently, tensile tests and DMA experiments were conducted to investigate the mechanical properties of the UPRs synthesized from different waste sources. Finally, based on DSC results, the curing process of the UPRs was analyzed, and the corresponding curing kinetic equations were established. The key conclusions are summarized below:

(1) The alcoholysis wastes from different alcoholysis systems were successfully synthesized to UPRs. The UPR synthesized from the EG alcoholysis system exhibited a lower molecular weight and higher thermal stability compared to those derived from PG and DEG systems. Furthermore, all three UPRs contained macromolecules with exceptionally high molecular weights, as evidenced by the GPC analysis.

(2) All synthesized UPRs demonstrated high toughness, with the minimum elongation at break reaching 46%, which was more than four times that of traditional UPR. However, the UPR derived from DEG, characterized by longer molecular chains, showed reduced crosslinking density, leading to a significant decrease in tensile strength and *T_g_*.

(3) UPR synthesized from oligomers exhibited a storage modulus comparable to that of traditional UPR under low-temperature conditions. However, its modulus decreased more sharply with increasing temperature, and phase transitions occurred at lower temperatures. These properties suggest potential suitability for applications requiring high deformation under low-strength conditions.

(4) Longer molecular chains in the synthesized UPRs resulted in a slower curing rate and higher activation energy. Furthermore, due to the heterogeneity of the products obtained from alcoholysis, the collision frequency of reactive species in the reaction system for the UPRs synthesized from EG and DEG was higher than that for the UPRs synthesized from PG.

This study successfully synthesized unsaturated polyester resins (UPRs) from oligomeric byproducts generated in diverse alcoholysis systems. The experimental results confirmed effective structural conversion of waste materials into UPRs, with distinct property profiles observed across different glycolytic systems. The findings propose a sustainable strategy to valorize unavoidable oligomeric byproducts in conventional PET alcoholysis, demonstrating potential for improving PET recycling efficiency.

## Figures and Tables

**Table 1:** Molecular weight calculation results.

	*M_n_*	*M_w_*	*M_z_*	PDI
UEG	1065	2480	5557	2.328
UPG	2345	5970	11,588	2.545
UDEG	1799	6593	15,006	3.664

**Table 2:** Calculation results of crosslinking density for UPRs.

Sample	*E_r_* (90 °C) (MPa)	*T_g_* (°C)	*v_e_* (mol/cm^3^)
UEG	3.554	68	0.392
UPG	0.902	53	0.100
UDEG	1.153	35	0.127

**Table 3:** *T_p_* and *P_w_* of UPRs at different heating rates.

Sample	5 °C/min		10 °C/min		15 °C/min		20 °C/min	
	*T_p_* (*K*)	*P_w_* (*K*)	*T_p_* (*K*)	*P_w_* (*K*)	*T_p_* (*K*)	*P_w_* (*K*)	*T_p_* (*K*)	*P_w_* (*K*)
UEG	392.34	28.69	401.20	33.21	407.30	34.66	417.69	31.74
UPG	384.72	33.91	394.04	32.81	400.33	37.78	405.01	36.89
UDEG	381.78	39.15	393.12	39.51	397.47	40.67	401.86	44.56

**Table 4:** Calculation results of SB model parameters for UPRs.

	ln*A*	*E*	*m*	*n*
UEG	26.150	81.339 KJ/mol	0.588	1.204
UPG	25.512	81.630 KJ/mol	0.416	1.340
UDEG	26.457	87.610 KJ/mol	0.222	1.232

## Data Availability

The original contributions presented in this study are included in the article. Further inquiries can be directed to the corresponding authors.
